# Life-Threatening Duodenal Perforation Following Endoscopic Retrograde Cholangiopancreatography in an Elderly Patient: A Case Report

**DOI:** 10.7759/cureus.98498

**Published:** 2025-12-05

**Authors:** Christina Chrysanthi Theocharidou, Fotini Ampatzidou, Savvas Simeonidis, Andreas Papadimos, Athina Lavrentieva

**Affiliations:** 1 Internal Medicine and Critical Care, General Hospital of Thessaloniki "G.Papanikolaou", Thessaloniki, GRC; 2 Critical Care, General Hospital of Thessaloniki "G.Papanikolaou", Thessaloniki, GRC; 3 Surgery, General Hospital of Thessaloniki "G.Papanikolaou", Thessaloniki, GRC; 4 Internal Medicine, General Hospital of Thessaloniki "G.Papanikolaou", Thessaloniki, GRC

**Keywords:** duodenal perforation, endoscopic retrograde cholangiopancreatography (ercp), intensive care unit, pancreatic-biliary cancer, pneumothorax

## Abstract

Endoscopic retrograde cholangiopancreatography (ERCP) is a valuable diagnostic and therapeutic procedure for pancreaticobiliary disorders, but may rarely result in severe complications such as duodenal perforation. A 77-year-old man with hypertension, diabetes, dyslipidemia, and benign prostatic hyperplasia presented with painless obstructive jaundice for a duration of four weeks. Laboratory tests showed a cholestatic pattern and elevated carbohydrate antigen 19-9. Magnetic resonance cholangiopancreatography (MRCP) demonstrated marked dilation of the common bile duct with abrupt distal tapering, mild pancreatic duct dilation, and a periampullary diverticulum, suggesting a periampullary neoplastic lesion or fibrotic stenosis. An ERCP was performed for diagnostic clarification and potential therapeutic decompression. Difficult cannulation required a precut sphincterotomy, after which a duodenal perforation was suspected. Post-procedure, the patient developed extensive subcutaneous, retroperitoneal, and mediastinal emphysema, pneumopericardium, and bilateral pneumothoraces. A CT confirmed free intraperitoneal air and contrast extravasation in the retroperitoneal space anterior to the left renal fascia, consistent with a perforation of the third portion of the duodenum. Emergency laparotomy identified and repaired the defect with a Graham patch. Despite intensive care with broad-spectrum antimicrobials and vasopressors, the patient developed septic shock and multi-organ failure and died nine days later. This case underscores the catastrophic potential of ERCP-related duodenal perforation and the importance of early CT diagnosis and individualized intervention in high-risk patients.

## Introduction

Endoscopic retrograde cholangiopancreatography (ERCP) is a cornerstone diagnostic and therapeutic procedure for managing a wide spectrum of biliary and pancreatic diseases. Although generally safe when performed by experienced endoscopists, it carries a recognized risk of complications, including pancreatitis, hemorrhage, infection, and perforation. Among these, duodenal perforation is one of the most serious and potentially fatal complications, with mortality rates reported as high as 18% [1].

The clinical presentation of ERCP-related perforation is often variable and nonspecific, ranging from mild abdominal discomfort to rapidly progressive, life-threatening peritonitis. The CT remains the diagnostic gold standard, allowing for the detection of extraluminal air, contrast leakage, and associated fluid collections that guide further management. Treatment options range from conservative management to urgent surgical intervention, depending on the type and extent of the perforation, the patient’s comorbidities, and hemodynamic status. Given the substantial morbidity and mortality associated with this complication, prompt recognition and timely management are essential [1-3]. This report aims to describe a rare and severe case of duodenal perforation following ERCP in an elderly patient, complicated by extensive retroperitoneal and thoracic air spread.

## Case presentation

A 77-year-old man with a medical history of arterial hypertension, type 2 diabetes mellitus, dyslipidemia, and benign prostatic hyperplasia presented with painless jaundice for four weeks. Laboratory findings at hospital admission (Table 1) demonstrate a cholestatic pattern with elevated direct bilirubin, liver enzymes, and carbohydrate antigen 19-9 (CA 19-9).

**Table 1:** Laboratory values at hospital admission (before ERCP) and on day 9 after ICU admission ERCP: Endoscopic retrograde cholangiopancreatography, CA 19-9: Carbohydrate antigen 19-9

Parameter	Admission	Day 9	Reference range
Alanine aminotransferase	229 U/L	830 U/L	<32 U/L
Aspartate aminotransferase	221 U/L	353 U/L	<33 U/L
Gamma-glutamyl transferase	662 U/L	567 U/L	5–36 U/L
Alkaline phosphatase	427 U/L	438 U/L	35–105 U/L
Total bilirubin	11.48 mg/dL	10.11 mg/dL	<1.2 mg/dL
Direct bilirubin	10.71 mg/dL	9.17 mg/dL	0–0.3 mg/dL
Albumin	2.87 g/dL	2.17 g/dL	3.5–5.2 g/dL
INR	1.4	2.1	0.8–1.1
CA 19-9	543 U/mL	—	<37 U/mL

**Figure 1:**
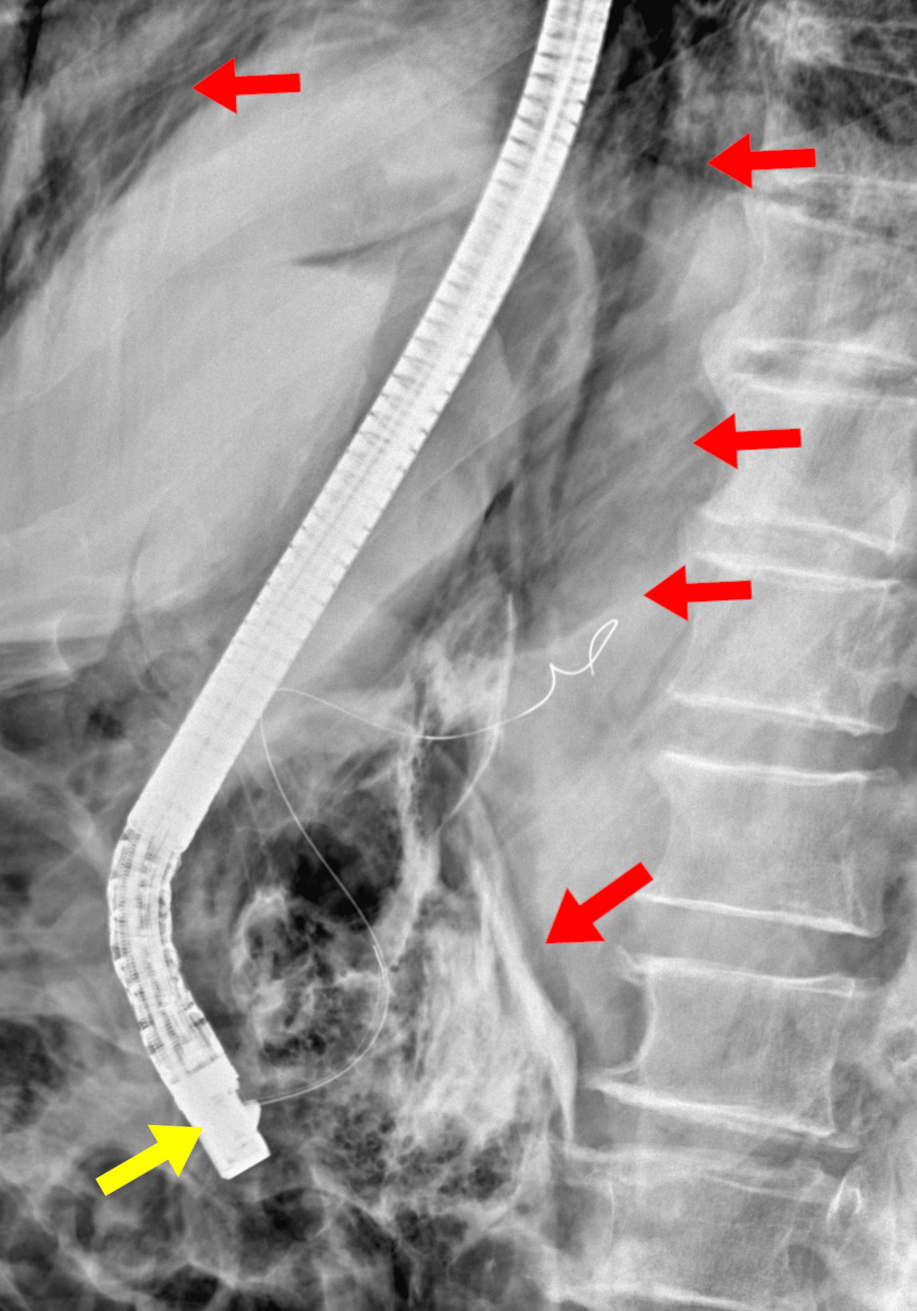
Image obtained during ERCP demonstrating the endoscope positioned in the duodenum (yellow arrow) Extensive extraluminal free air (red arrows) is visible within the abdominal cavity, consistent with post-ERCP perforation and pneumoperitoneum. ERCP: endoscopic retrograde cholangiopancreatography

Magnetic resonance cholangiopancreatography (MRCP) demonstrated marked dilation of the intra- and extrahepatic bile ducts, with the common bile duct measuring 25 mm and an abrupt distal tapering at the level of the ampulla. The main pancreatic duct was mildly dilated (6.6 mm), and a small periampullary diverticulum was identified. The overall findings were consistent with distal biliary obstruction and suggested either a periampullary neoplastic lesion or fibrotic stenosis. An ERCP was performed as the next diagnostic and therapeutic step. The major papilla appeared normal with mild erythema, but cannulation proved difficult, necessitating a precut sphincterotomy. A duodenal perforation was suspected intra-procedurally (Figure 1).

**Figure 2:**
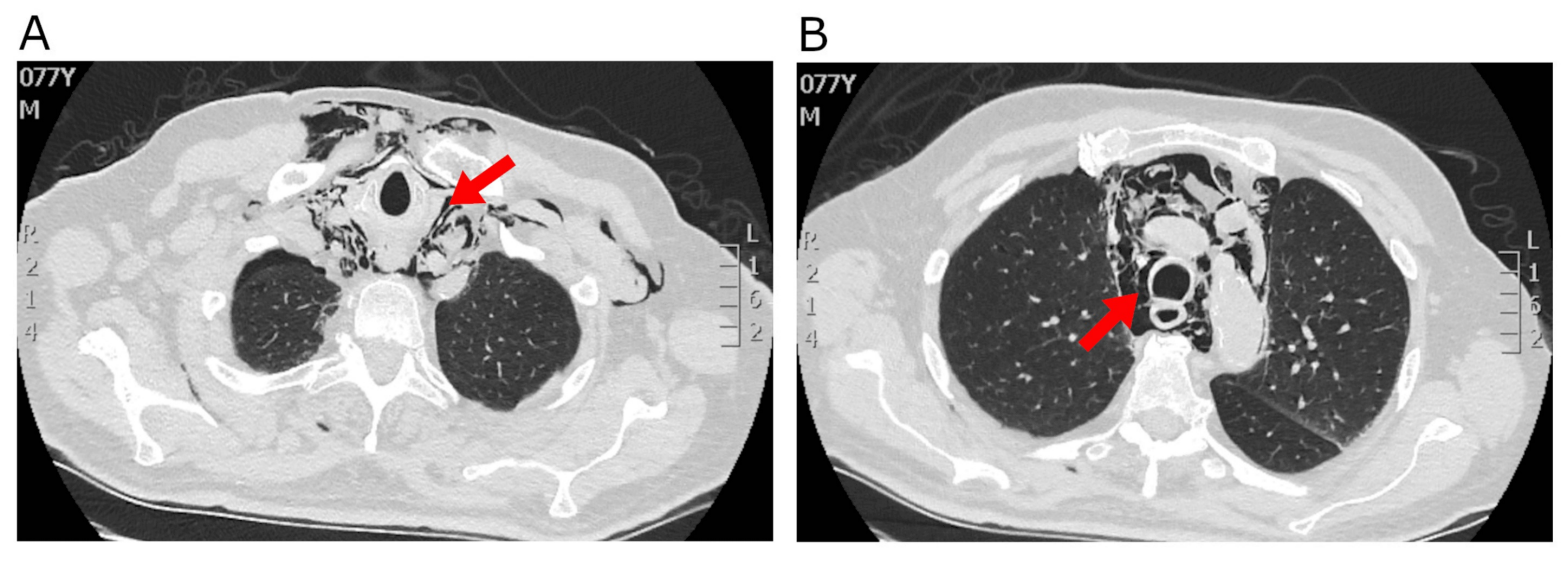
CT of the chest showing pneumomediastinum (red arrows) of a large extent (A and B)

**Figure 3:**
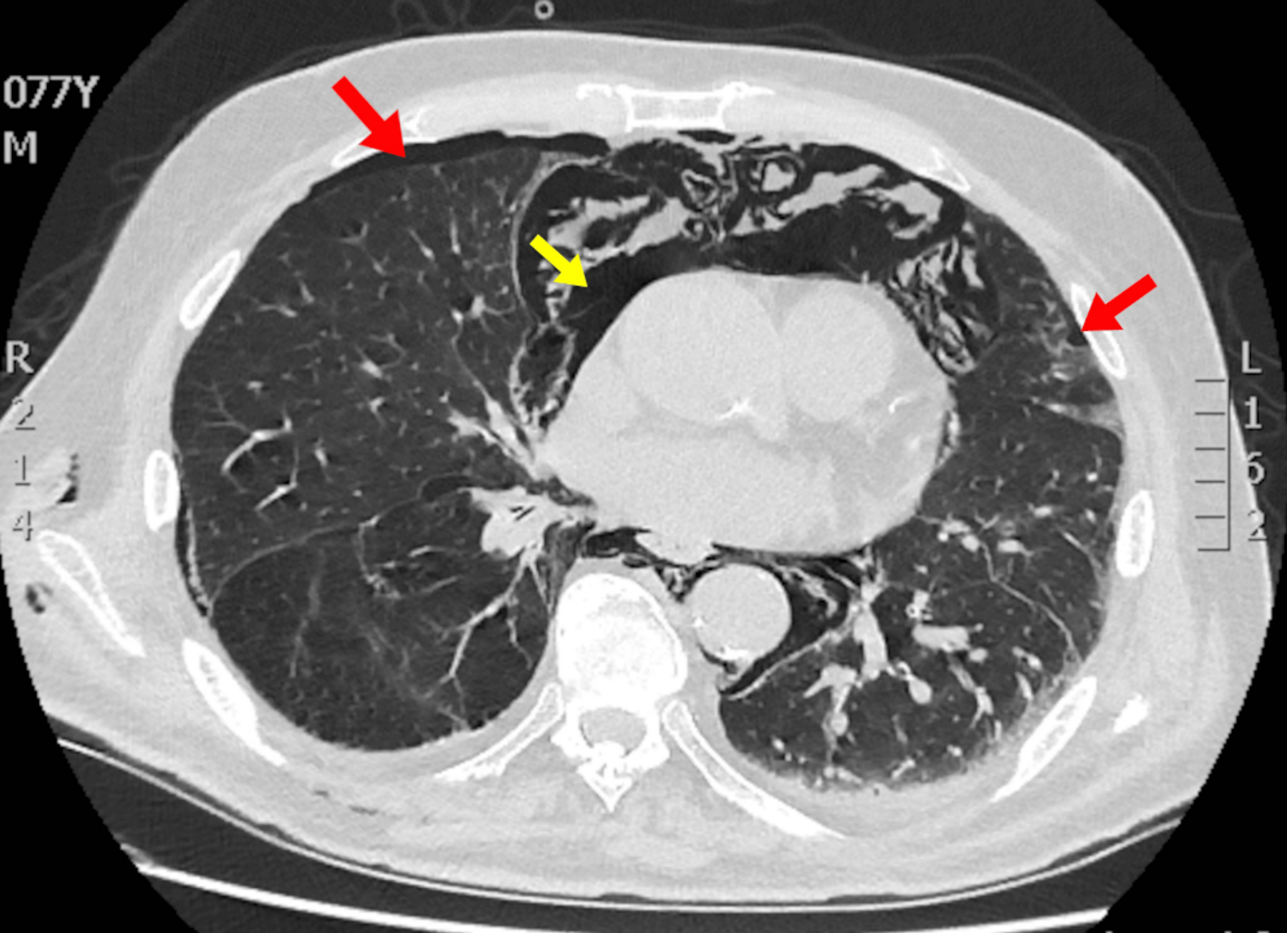
CT of the chest demonstrating pneumopericardium (yellow arrow) and small pneumothoraces (red arrows)

**Figure 4:**
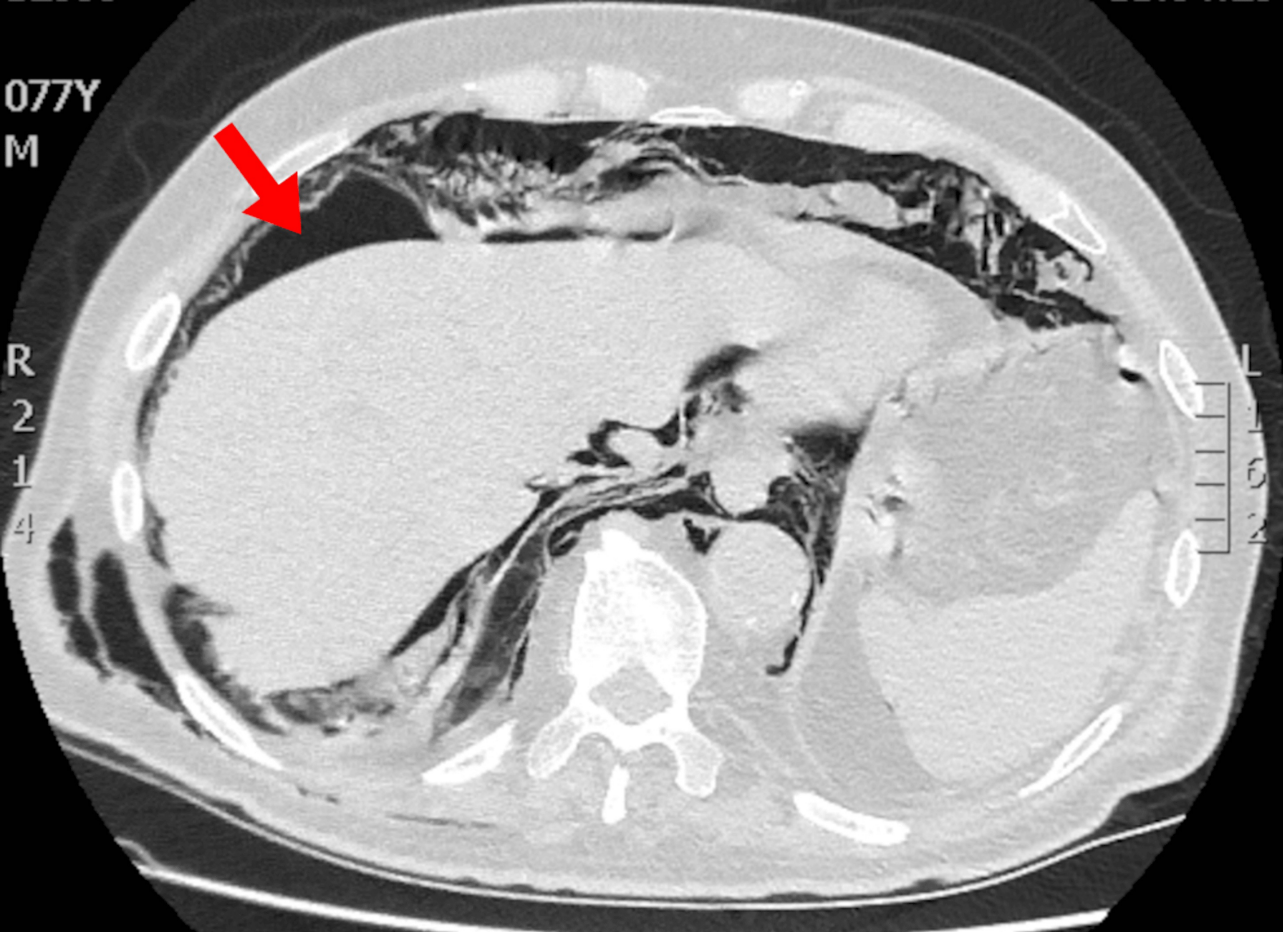
CT of the abdomen showing significant free intraperitoneal air (red arrow points to the pneumoperitoneum)

Post-procedure, the patient developed facial subcutaneous emphysema and right upper quadrant pain. The CT revealed extensive subcutaneous emphysema extending from the face to the inguinal region, retroperitoneal and mediastinal emphysema, pneumopericardium, and small bilateral pneumothoraces (Figures 2-3). Abdominal CT also demonstrated free intraperitoneal air, confirming the presence of perforation (Figure 4). Contrast material from the ERCP was noted inside the lumen of the duodenum, extending up to the proximal jejunum. Additionally, pooling of contrast medium was noted outside the intestinal lumen, collecting in the retroperitoneal space anterior to the left renal fascia. The contrast was in contact with the third portion of the duodenum, suggesting that the leakage likely originates from a perforation in this segment.

An emergency laparotomy confirmed perforation in the third part of the duodenum. Primary repair with a Graham patch was performed, along with cholecystectomy, feeding jejunostomy, decompressive gastrostomy, and placement of subhepatic and pelvic drains. Bilateral chest tubes were placed for the pneumothoraces.

Postoperatively, sedation, analgesia, and mechanical ventilation were initiated with ventilator settings of fraction of inspired oxygen (FiO₂) 50% and positive end-expiratory pressure (PEEP) 5 cmH₂O. Arterial blood gas analysis showed a partial pressure of oxygen (PaO₂) of 120 mmHg. Lung ultrasound showed present bilateral lung sliding with no evidence of pneumothorax. The patient had an acute physiology and chronic health evaluation (APACHE) II score of 18 and a sequential organ failure assessment (SOFA) score of 10. Broad-spectrum antimicrobials (ceftolozane-tazobactam, tigecycline, and anidulafungin) and vasopressor support, using norepinephrine at 0.9 mcg/kg/min, were initiated.

The patient’s condition progressively deteriorated despite maximal supportive therapy. Over the ensuing days, he developed worsening septic shock with increasing vasopressor requirements and evolving multi-organ dysfunction. Follow-up CT imaging showed no new findings. By day six, he required norepinephrine at 1.5 mcg/kg/min and vasopressin at 0.03 U/min; he became anuric and required continuous renal replacement therapy (CRRT). On day nine, laboratory findings reflected severe systemic inflammation, with marked leukocytosis (WBC 33.4 × 10⁹/L, with 87% neutrophils) and elevated procalcitonin (8.27 ng/mL, reference range <0.05 ng/mL). Direct bilirubin and liver enzymes remained elevated (Table 1). Despite broad-spectrum antimicrobials, vasopressor support, and CRRT, the patient continued to worsen and died nine days after ICU admission due to refractory septic shock and multi-organ failure.

## Discussion

Endoscopic retrograde cholangiopancreatography is a widely used diagnostic and therapeutic procedure for biliary and pancreatic diseases. Despite its established role, it carries a small but significant risk of complications, some of which can be life-threatening. Among these, duodenal perforation is one of the most serious, with an incidence of 0.1% to 1% and a reported mortality rate of 16% to 18% [1,4-6]. We present such a fatal case with extensive air dissemination and a rare clinical presentation of subcutaneous emphysema from the face to the inguinal region.

Mechanisms and classification

Perforation during ERCP can occur through several mechanisms [2,3]. Passage of the endoscope may cause direct trauma to the duodenal wall, resulting in an intraperitoneal perforation. Endoscopic sphincterotomy is the most common cause, responsible for approximately 40% of cases, especially when the incision extends beyond the intramural portion of the bile or pancreatic duct [3,7]. Endoscope manipulation accounts for around 25% of perforations, while guidewire-related injuries represent about 15% and are usually minor. Other instruments, such as dilators, baskets, or stents, are less frequent causes [3]. The classification proposed by Stapfer et al. remains the most widely used [4]: type I are lateral or medial duodenal wall perforations, usually intraperitoneal, caused by the endoscope, which typically require immediate surgery; type II are periampullary injuries, often related to sphincterotomy or precut procedures, usually retroperitoneal leaks, which may initially be managed conservatively; type III are distal bile duct injuries, often due to guidewires or baskets, generally small and non-operative; and type IV is retroperitoneal air without contrast leak, likely due to microperforations or excessive insufflation, and is treated conservatively.

This classification correlates well with both the mechanism and severity of injury, guiding appropriate management. In our patient, a type I perforation of the third portion of the duodenum was identified, likely resulting from direct mechanical trauma during difficult cannulation and precut sphincterotomy. The injury produced a large retroperitoneal defect with extensive air spread into the mediastinum and pleural cavities, consistent with pneumomediastinum, pneumopericardium, and bilateral pneumothoraces. The defect required emergency surgical repair with a Graham patch, in line with current recommendations for type I injuries.

Risk factors and diagnosis

Recognized risk factors include altered postsurgical anatomy, periampullary diverticula, papillary stenosis, sphincter of Oddi dysfunction, biliary strictures, and prolonged or technically difficult procedures. Precut sphincterotomy and repeated cannulation attempts are also independent risk factors [8-10]. In our patient, several of these risk factors were present. A periampullary diverticulum was demonstrated on MRCP, and biliary cannulation was difficult, requiring a precut sphincterotomy. Together with the patient’s advanced age and comorbidities, these factors likely contributed to the occurrence and severity of duodenal perforation and the unusually extensive spread of air.

Clinical diagnosis in the early stages can be challenging, as symptoms are often nonspecific. Patients may present with abdominal pain, distension, or subcutaneous emphysema, but peritonitis is not always evident, particularly in retroperitoneal perforations [8]. Imaging is therefore crucial. In our case, fluoroscopy during ERCP already showed extensive extraluminal air, raising immediate suspicion of perforation. Abdominal CT scan is the diagnostic modality of choice, as it was with this case, allowing detection of free intraperitoneal or retroperitoneal air, contrast extravasation, and associated fluid collections [11,12].

Thoracic complications

Rarely, air may disseminate from the retroperitoneum into the thoracic cavity, leading to pneumothorax, pneumomediastinum, or pneumopericardium [13-15]. This occurs when insufflated air tracks along fascial planes through the diaphragmatic hiatuses [10,16]. Although uncommon, pneumothorax can rapidly cause respiratory compromise and often coexists with subcutaneous emphysema and retroperitoneal air [10,13,14]. Pneumopericardium, though rare, can progress to cardiac tamponade, requiring prompt recognition and sometimes invasive drainage [17]. In our patient, all three complications were present, i.e., bilateral pneumothoraces, extensive pneumomediastinum, and pneumopericardium, along with a marked subcutaneous emphysema from the face to the inguinal region. These findings illustrate the potential for massive air dissemination across fascial planes following duodenal perforation and highlight the importance of early imaging and close cardiopulmonary monitoring in such cases.

Prognosis and clinical implications

The ERCP-related perforations, though rare, carry high morbidity and mortality. The coexistence of thoracic complications such as pneumothorax or pneumopericardium indicates extensive extraluminal air spread and typically reflects a more severe underlying injury. In our case, the combination of advanced age, comorbidities, periampullary diverticulum, difficult cannulation, and precut sphincterotomy likely increased procedural risk, while the retroperitoneal and thoracic air dissemination contributed to a fatal outcome despite prompt surgical repair and intensive management. Early recognition, rapid surgical intervention, and multidisciplinary care remain critical in ERCP-related duodenal perforations, particularly in elderly and high-risk patients.

## Conclusions

Duodenal perforation following ERCP is a rare but potentially fatal complication, particularly in elderly patients with multiple comorbidities and anatomical risk factors such as periampullary diverticula. Difficult cannulation and precut sphincterotomy further increase the risk of injury. Early recognition and prompt, individualized management are essential to improving outcomes. However, despite advances in endoscopic techniques, surgical management, and critical care, mortality remains substantial, reflecting both the severity of this complication and the vulnerability of affected patients.
